# Supplementary material for: A Fiber-Optic Sensor-Embedded and Machine Learning Assisted Smart Helmet for Multi-Variable Blunt Force Impact Sensing in Real Time
Source: Biosensors (Basel). 2022 Dec 13;12(12):1159. doi: 10.3390/bios12121159 (PMC9775411; doi:10.3390/bios12121159)
Supplement: Supplementary file 1 [file biosensors-12-01159-s001.zip › biosensors-1989832-supplementary.pdf]

SUPPLEMENTARY INFORMATION

A Fiber-Optic Sensor-Embedded and Machine Learning Assisted Smart Helmet for Multi-Variable Blunt Force Impact Sensing in Real Time

Yiyang Zhuang [1,2], Taihao Han [3], Qingbo Yang [4], Ryan O'Malley [2], Aditya Kumar [3], Rex E. Gerald II [2] and Jie Huang [2,*]

[1] Research Center for Optical Fiber Sensing, Zhejiang Laboratory, Hangzhou 311121, China

[2] Department of Electrical and Computer Engineering, Missouri University of Science and Technology, Rolla, MO 65409, USA

[3] Department of Materials Science and Engineering, Missouri University of Science and Technology, Rolla, MO 65409, USA

[4] Cooperative Research, College of Agriculture, Environmental and Human Sciences, Lincoln University of Missouri, Jefferson City, MO 65102, USA

*Corresponding author

Jie Huang (jieh@mst.edu)

S1. Basic introduction to the ML models used in this study

a. Support Vector Machine (SVM)

SVM predicts new patterns based on the training data as the goal of learning a maximum-margin hyperplane in the feature space [17]. The maximum hyperplane is formed when the decision boundary has the maximal distance from any training data. During the training step, input variables are mapped from a low-dimension to a high-dimension feature space via kernel functions. The SVM attempts to determine a set of linear or nonlinear (e.g., polynomial or sigmoidal) objective functions that have a maximum deviation of ε with respect to the actual values in the training dataset.

b. Gaussian Process Regression (GPR)

Gaussian process regression (GPR) applies a stochastic process to collect random variables, any finite number of which have a joint Gaussian distribution [18]. GPR organizes data in a manner, based on a non-parametric approach, such that any given subset of the organized data invariably follows a multivariate Gaussian distribution.

c. Random Forest (RF)

Random Forest, a collection of tree predictors, is based on the integration of two machine learning techniques: bagging and random feature selection [19, 20]. During the training process, a series of "deep" unpruned decision trees are grown, thus dividing the entire training dataset into multiple, uncorrelated splits. For any given input, the RF model collects predictions from all of its constituent trees and subsequently averages them to yield the final prediction.

d. K-nearest Neighbor Instance-Based Learner (IBK)

K-nearest neighbor instance-based learning (IBK), a lazy learner, collects the training data as instances and find the closest k nearest training records via distance measurements [21]. In order to compute the testing records, the model finds the instances which have the most similar input

variables to the testing data in the collection and returns outputs of the instances as the predicted value for testing data [22]. For multiple attributes, the instance is classified by a majority vote of the k nearest neighbors. The weights of each instance are weighted by the inverse of the attribute distance from the testing data, which allows the model to modify the strength of the votes via the distance from testing data [23]. This method was used because it has several advantages [21]: (1) simplicity- a rigorous analysis can be used in the model; (2) robust model- noise and irrelevant attributes can be tolerated; (3) relaxed concept bias- piecewise linear approximation of concepts are learned; (4) low updating cost- saves the new instance without additional computation. The only limitation of this model is that the prediction accuracy depends on the ability to store instances.

e. Ensemble model: Additive Regression-Support Vector Machine (AR-SVM)

The additive regression (AR) technique employs the gradient boosting approach to improve the prediction performance of the SVM. In the first step, the standalone SVM model is employed, whilst using the entire database, with all input and output variables, for construction of "deep" trees. In the second step, residuals of the predictions (i.e., the differences between actual and SVM-predicted values) are used to construct a second set of trees; the objective, here, is to train the second set of trees to fit the residuals such that the overall training error is reduced. This tandem between the aforementioned pair of steps—of performing predictions using the SVM-model, and subsequently refining the prediction performance by fitting the residuals—is repeated over several iterations until convergence is reached, that is, reduction in training error is $< 10^{-6}$ units for 3 successive iterations. In the last step, the predictions of all trees within the ensemble are averaged to obtain the final predictions.

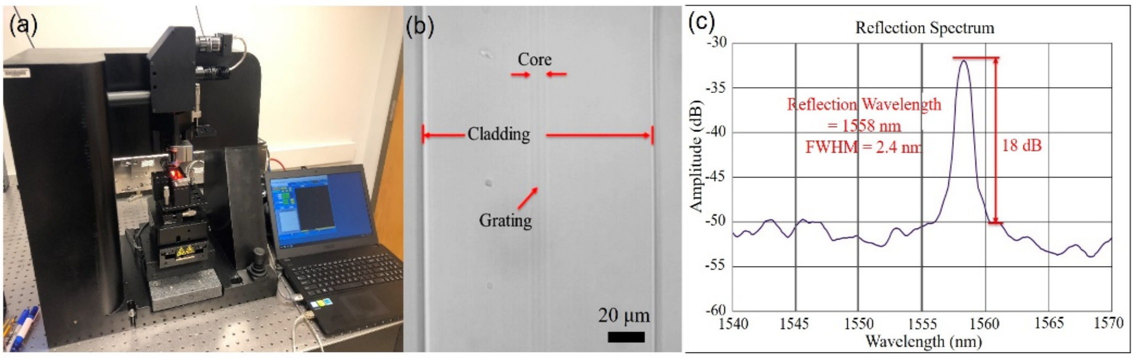

Figure S1. The Newport *femto*second laser microfabrication system, an FBG structure, and the corresponding reflection spectrum. (a) The newly purchased *fs* laser microfabrication system in the Missouri S&T Blast Lab (Emerson Electric. Co. Hall, room G-18). (b) A newly made FBG structure observed under a microscope with annotated fiber structures. The fabricated fiber Bragg grating is located within the fiber core. The scale bar is 20 μm. (c) The reflection spectrum collected using the fabricated FBG structure sample in (b).

S2. Brief steps of FBG sensor fabrication using the *femto*second laser microfabrication system

a. *Sample installation*: Fabrication of the FBG sensor starts with the immobilization of a single-mode optical fiber mounted on top of a microscopic slide, which is then fixed to the micro-fabrication stage. Next, a couple of drops of RI matching gel are applied atop the optical fiber. Next, another slide is quickly placed on top of the fiber, so that a sandwich structure is formed, with all parts affixed to the stage.

b. *Optical fiber alignment and focal plane adjustment*: To obtain and retain a clear view of the fiber core during fabrication of the FBG, a whole region of interest of the fiber needs to be aligned perpendicular to the laser path using the X/Y stages and the rotating and tilting micrometers. Meanwhile, the focal plane of the laser-guiding lens must be adjusted such that the fiber core

boundary is blurred; the boundary, however, must become apparent again when moving the z stage up or down by 5 μm.

c. *Parameter designations*: The featured wavelength and bandwidth of the fabricated FBG can be changed by controlling the repetition rate of the *fs* laser and the speed of the moving stage. Here, the wavelength, bandwidth, and reflectivity of the FBG are determined by:

$$\lambda_{FBG} = \frac{2nv_{trans}}{mf_r} \tag{S1}$$

$$\Delta\lambda = \frac{\lambda_{FBG}^2}{2n} \cdot \frac{1}{L}\sqrt{\left(\frac{\Delta nL}{\lambda_{FBG}}\right)^2 + 1} \tag{S2}$$

$$R = \tanh^2\left(\frac{L\pi}{\lambda_{FBG}}\Delta n\right) \tag{S3}$$

where $\boldsymbol{v_{trans}}$ is the moving speed of the laser head along the Y-axis, $\boldsymbol{m}$ is the order of the FBG, $\boldsymbol{f_r}$ is the *fs* laser pulse repetition frequency, $\boldsymbol{L}$ is the length of FBG, and $\boldsymbol{\Delta n}$ is the RI perturbation inside the optical fiber core. From Equations (S2) and (S3), we can calculate that the longer the length of the FBG, narrower the bandwidth and the higher the reflectivity. Hence, the FBG bandwidth can be reduced by decreasing $\boldsymbol{\Delta n}$ or using a higher order of $\boldsymbol{m}$. In addition, the side lobes of the FBG can be suppressed by making an apodized FBG instead of the regular FBG. The apodized profile can also be modified by changing the laser power.

d. *Linear single-line raster*. In the final step, the laser writing position along the fiber core is progressively changed until a sufficient number of structural units are fabricated for an FBG.

A microscopic image of a single FBG structure, made along an optical fiber core using the *femto*FBG, is shown in **Figure 2b**. The boundary between the core and cladding can be observed.

The dark line along the center of the core is the FBG pattern that was fabricated. This result demonstrates our ability to fabricate an FBG—using the state-of-the-art microfabrication system in the Missouri S&T Blast Lab—without compromising the fiber polymer cladding. Through our experiments, we have also finalized the "optimal" set of parameters for fabrication: stage translation speed = 95 µm/s; repetition rate of the *fs* laser = 61 Hz; and, the RI profile set to a Gaussian distribution. The reflection spectrum obtained from the FBG is shown in **Figure 2c**. The figure exemplifies a high-fidelity result that can only be obtained if the FBG structure is accurate. The peak observed at 1558 nm is a clear sign that the FBG is fully functional; notably, its full-width-half-maximum (FWHM) bandwidth is 2.4 nm and the signal-to-noise ratio is 18 dB, which also corroborate the FBG's reasonable performance.

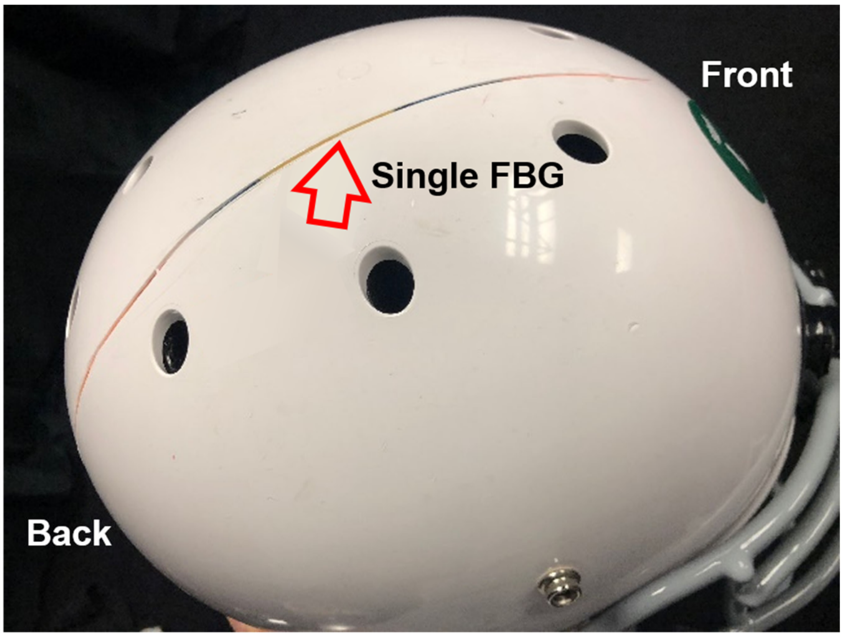

Figure S2 Picture of a single FBG embedded smart helmet prototype. Red arrow shows the location of the FBG sensor, which is fabricated with a length of 0.5 cm.

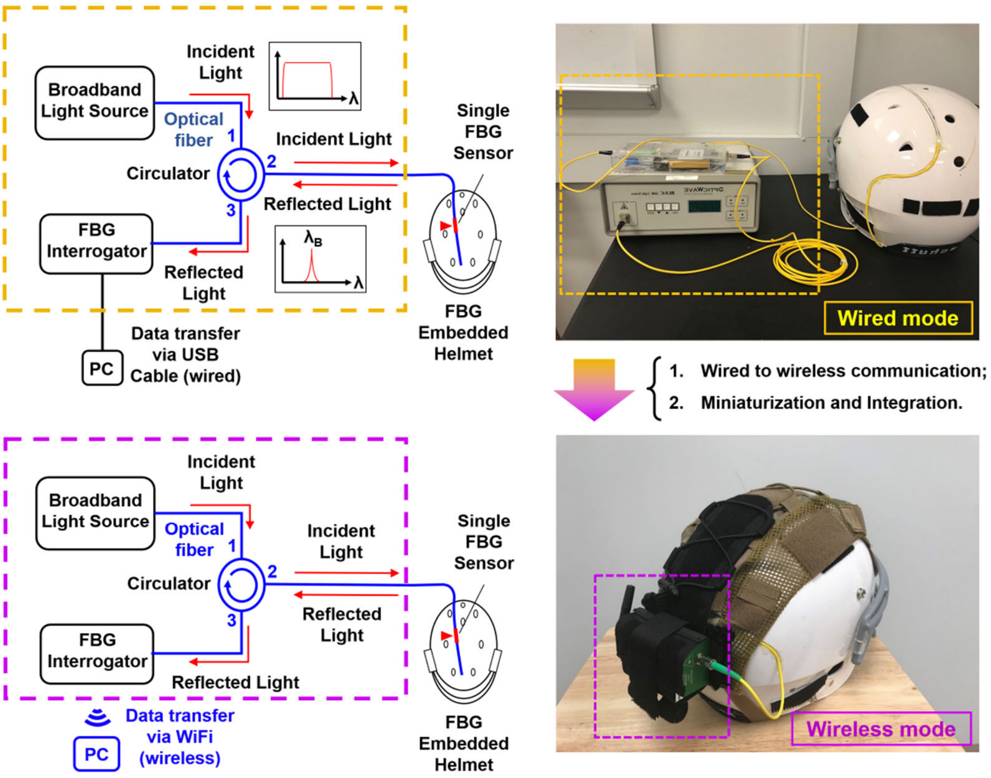

Figure S3 Schematics of the wired (upper) and wireless (lower) interrogation methods.

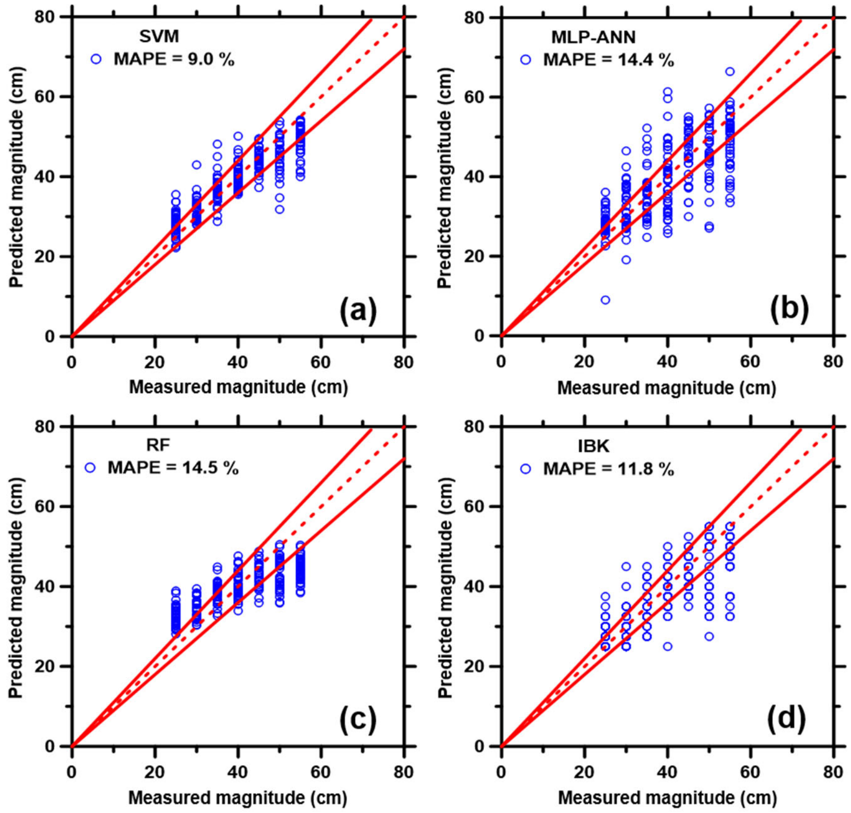

Figure S4. Graphs of predicted magnitude (bowling ball initial release height in cm) versus measured magnitude derived from four ML models. The ML models employed include: (a) Support Vector Machine (SVM), (b) Multilayer Perceptron—Artificial Neural Network (MLP-ANN), (c) Random Forest (RF), (d) IBK. The plotted data represent 25% of the parent Database that were not previously included in the training process of the ML models. The dashed line represents the line of ideality and the solid lines represent ±10% boundaries.

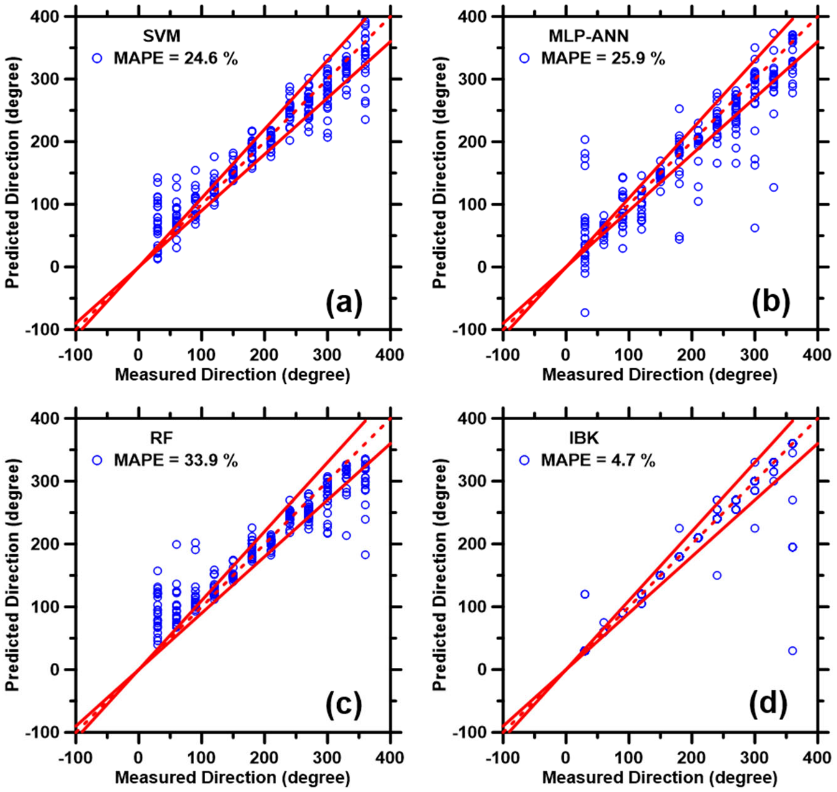

Figure S5. Graphs of predicted direction versus measured direction derived from four ML models. The ML models employed include: (a) Support Vector Machine (SVM), (b) Multilayer Perceptron—Artificial Neural Network (MLP-ANN), (c) Random Forest (RF), (d) IBK. The plotted data represent 25% of the parent Database that were not previously included in the training process of the ML models. The dashed line represents the line of ideality and the solid lines represent ±10% boundaries.

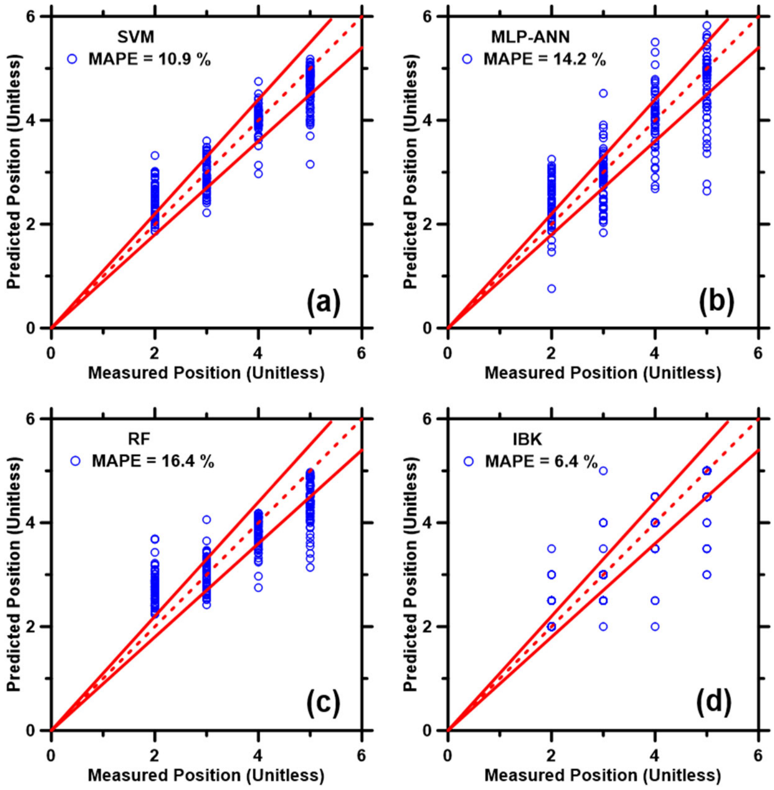

Figure S6. Graphs of predicted impact latitude (position) versus measured latitude derived from four ML models. The ML models employed include: (a) Support Vector Machine (SVM), (b) Multilayer Perceptron—Artificial Neural Network (MLP-ANN), (c) Random Forest (RF), (d) IBK. The plotted data represent 25% of the parent Database that were not previously included in the training process of the ML models. The dashed line represents the line of ideality and the solid lines represent ±10% boundaries.

Table S1. Prediction performance of ML models on impact magnitude based on the test set (i.e., 25% of the parent database). Five statistical parameters (i.e. R, R^2, MAE, MAPE, and RMSE) and the composite performance index (CPI) are shown.

ML Model	R<br>Unitless	R^2<br>Unitless	MAE<br>cm	MAPE<br>%	RMSE<br>cm	CPI<br>Unitless
**SVM**	**0.891**	**0.793**	**3.492**	**8.991**	**4.730**	**0.000**
RF	0.799	0.638	5.484	14.549	6.692	0.768
MLP-ANN	0.729	0.532	5.625	14.440	7.364	0.996
IBK	0.782	0.611	4.780	11.814	6.561	0.635

Table S2. Prediction performance of ML models on impact direction based on the test set (i.e., 25% of the parent Database). Five statistical parameters (i.e. R, R^2, MAE, MAPE, and RMSE) and the composite performance index (CPI) are shown.

ML Model	R<br>Unitless	R^2<br>Unitless	MAE<br>Degree	MAPE<br>%	RMSE<br>degree	CPI<br>Unitless
SVM	0.960	0.921	21.415	24.554	30.781	0.282
RF	0.948	0.899	26.528	33.883	39.360	0.652
MLP-ANN	0.918	0.843	25.811	25.888	43.114	0.938
**IBK**	**0.952**	**0.905**	**7.492**	**4.670**	**32.586**	**0.108**

Table S3. Prediction performance of ML models on impact latitude based on the test set (i.e., 25% of the parent Database). Five statistical parameters (i.e. R, R^2, MAE, MAPE, and RMSE) and the composite performance index (CPI) are shown.

ML Model	R	R^2	MAE	MAPE	RMSE	CPI
	Unitless	Unitless	Unitless	%	Unitless	Unitless
SVM	0.924	0.855	0.323	10.853	0.440	0.175
RF	0.864	0.746	0.481	16.445	0.613	0.887
MLP-ANN	0.842	0.708	0.440	14.169	0.621	0.926
**IBK**	**0.915**	**0.837**	**0.204**	**6.373**	**0.454**	**0.061**

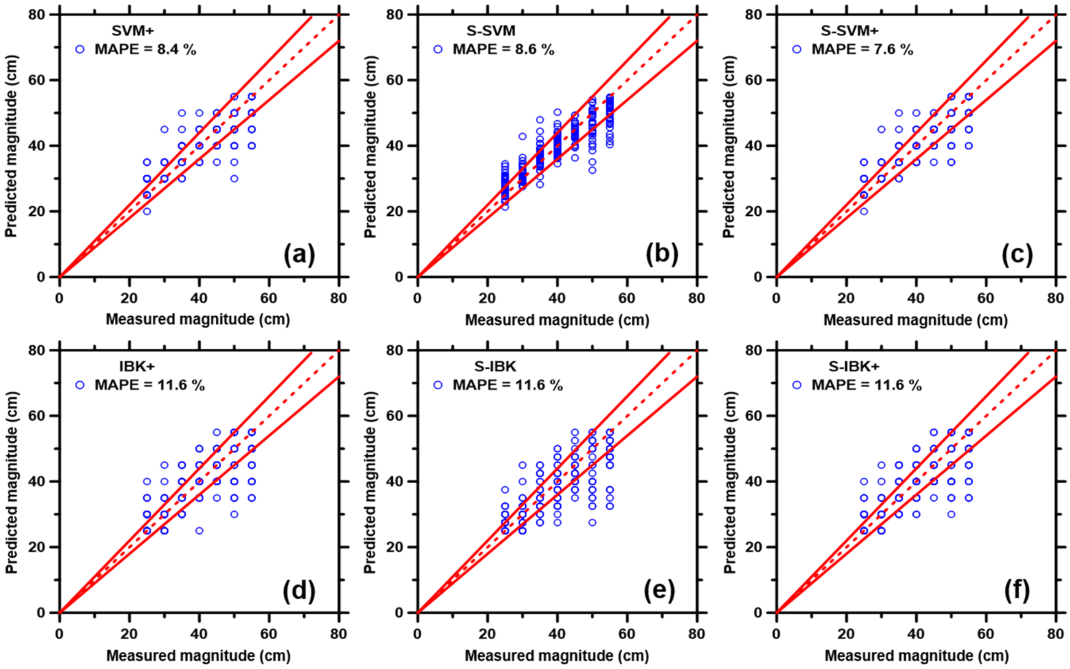

Figure S7 Graphs of predicted impact magnitude versus measured magnitude from six boosted ML models. The ML models employed include: (a) SVM+, (b) S-SVM, (c) S-SVM+, (d) IBK+, (e) S-IBK, (f) S-IBK+. The plotted data represent 25% of the parent database that were not previously included in the training process of the ML models. The dashed line represents the line of ideality and the solid lines represent ±10% boundaries.

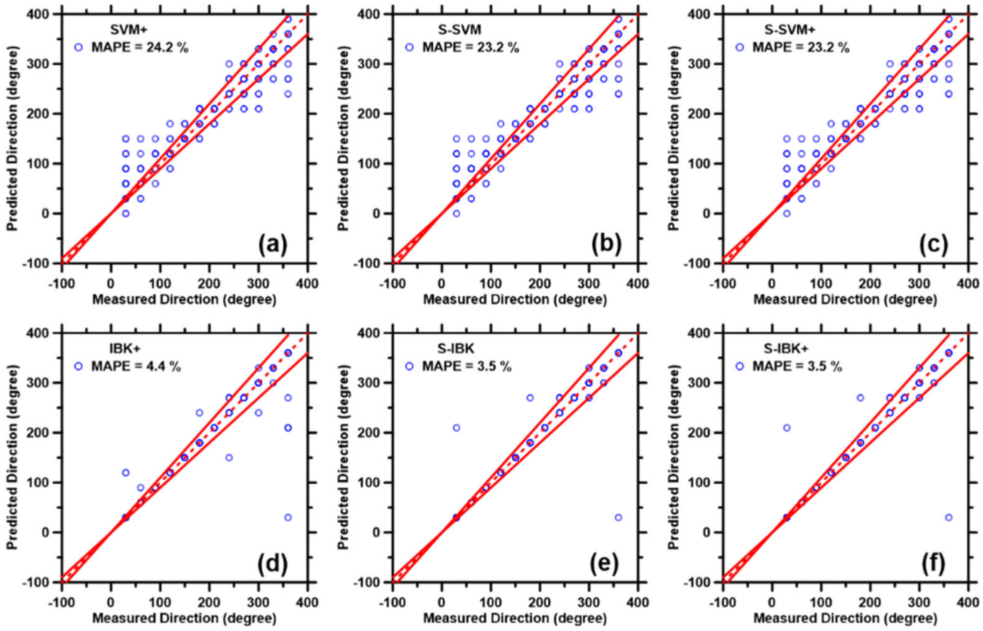

Figure S8 Graphs of predicted impact direction versus measured direction from six boosted ML models. The ML models employed include: (a) SVM+, (b) S-SVM, (c) S-SVM+, (d) IBK+, (e) S-IBK, (f) S-IBK+. The plotted data represent 25% of the parent database that were not previously included in the training process of the ML models. The dashed line represents the line of ideality and the solid lines represent ±10% boundaries.

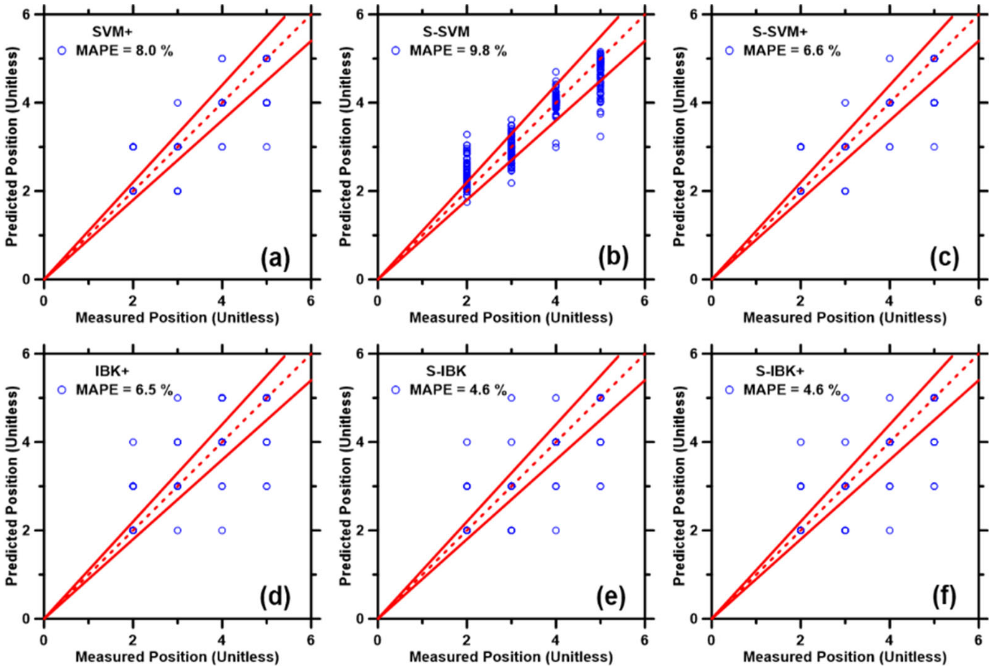

Figure S9 Graphs of predicted impact latitude versus measured latitude from six boosted ML models. The ML models employed include: (a) SVM+, (b) S-SVM, (c) S-SVM+, (d) IBK+, (e) S-IBK, (f) S-IBK+. The plotted data represent 25% of the parent database that were not previously included in the training process of the ML models. The dashed line represents the line of ideality and the solid lines represent ±10% boundaries.

Table S4. Prediction performance of boosted ML models on impact magnitude based on the test set (i.e., 25% of the parent Database). Five statistical parameters (i.e. R, R^2, MAE, MAPE, and RMSE) and the composite performance index (CPI) are shown.

ML Model	R<br>Unitless	R^2<br>Unitless	MAE<br>cm	MAPE<br>%	RMSE<br>cm	CPI<br>Unitless
SVM	0.891	0.793	3.492	8.991	4.730	0.162
SVM+	0.876	0.767	3.240	8.366	4.940	0.175
S-SVM	0.897	0.805	3.337	8.610	4.552	0.086
**S-SVM+**	**0.889**	**0.790**	**2.980**	**7.635**	**4.680**	**0.042**
IBK	0.782	0.611	4.780	11.814	6.561	0.981
IBK+	0.776	0.602	4.560	11.648	6.403	0.952
S-IBK	0.786	0.618	4.720	11.600	6.492	0.944
S-IBK+	0.782	0.611	4.540	11.570	6.332	0.921

Table S5. Prediction performance of boosted ML models on impact direction based on the test set (i.e., 25% of the parent Database). Five statistical parameters (i.e. R, R^2, MAE, MAPE, and RMSE) and the composite performance index (CPI) are shown.

ML Model	R<br>Unitless	R^2<br>Unitless	MAE<br>degree	MAPE<br>%	RMSE<br>degree	CPI<br>Unitless
SVM	0.960	0.921	21.415	24.554	30.781	0.801
SVM+	0.954	0.910	20.280	24.150	32.367	0.939
S-SVM	0.963	0.928	20.302	23.200	29.606	0.664
S-SVM+	0.956	0.913	19.920	23.154	32.087	0.886
IBK	0.952	0.905	7.492	4.670	32.586	0.654
IBK+	0.955	0.912	6.600	4.423	31.350	0.537
**S-IBK**	**0.971**	**0.942**	**3.960**	**3.536**	**25.385**	**0.000**
**S-IBK+**	**0.971**	**0.942**	**3.960**	**3.536**	**25.385**	**0.000**

Table S6. Prediction performance of boosted ML models on impact latitude based on the test set (i.e., 25% of the parent Database). Five statistical parameters (i.e. R, R^2, MAE, MAPE, and RMSE) and the composite performance index (CPI) are shown.

ML Model	R<br>Unitless	R^2<br>Unitless	MAE<br>Unitless	MAPE<br>%	RMSE<br>Unitless	CPI<br>Unitless
SVM	0.924	0.855	0.323	10.853	0.440	0.814
SVM+	0.901	7.960	0.228	7.960	0.486	0.647
S-SVM	0.934	0.871	0.292	9.750	0.414	0.599
S-SVM+	0.917	0.842	0.192	6.633	0.447	0.540
IBK	0.915	0.837	0.204	6.373	0.454	0.579
IBK+	0.909	0.827	0.184	6.453	0.473	0.645
**S-IBK**	**0.927**	**0.859**	**0.140**	**4.600**	**0.424**	**0.270**
**S-IBK+**	**0.927**	**0.859**	**0.140**	**4.600**	**0.424**	**0.270**

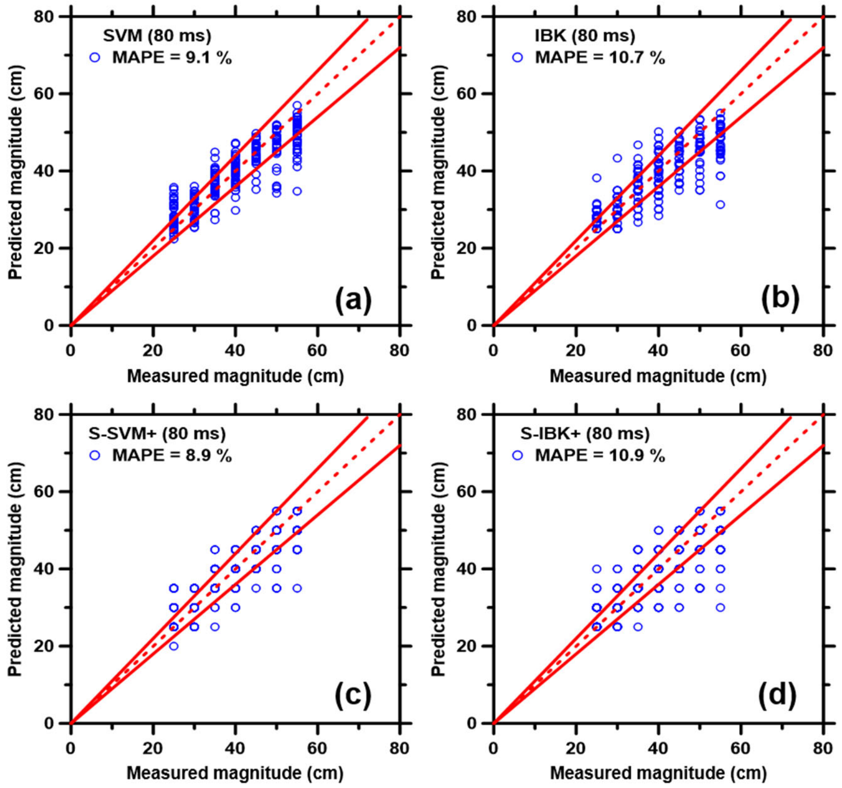

Figure S10 Graphs of predicted impact magnitude versus measured magnitude for the new training dataset with an 80 ms time frame. The ML models employed include: (a) SVM, (b) IBK, (c) S-SVM+, (d) S-IBK+. The plotted data represent 25% of the parent database that were not previously included in the training process of the ML models. The dashed line represents the line of ideality and the solid lines represent ±10% boundaries.

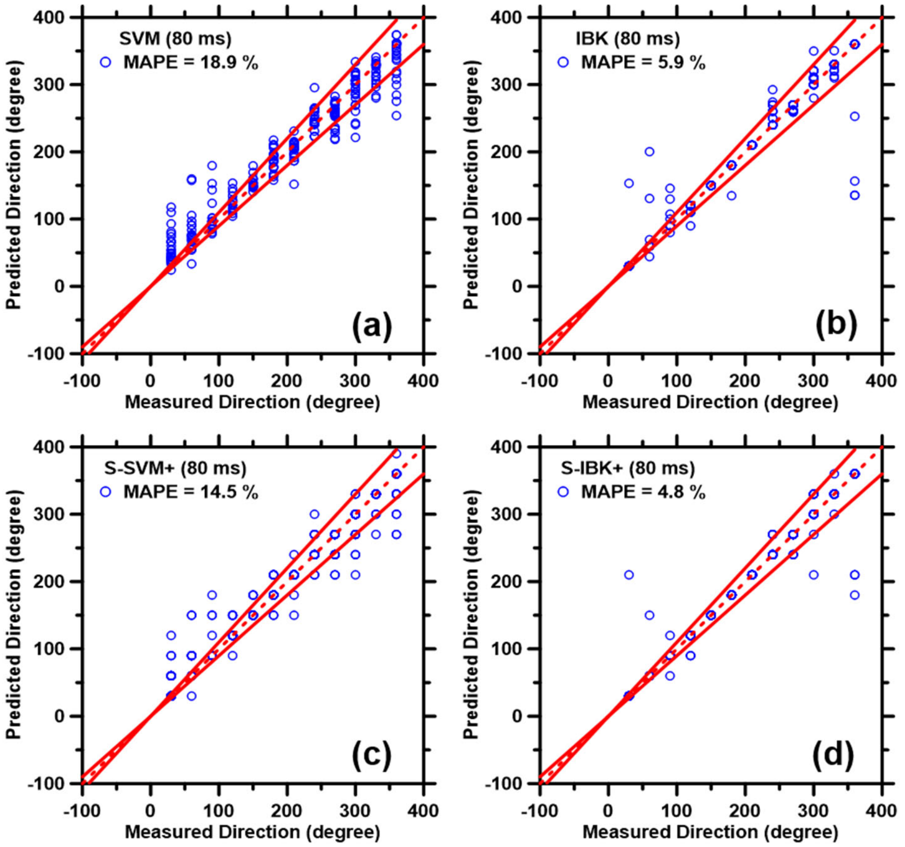

Figure S11 Graphs of predicted impact direction versus measured direction for the new training dataset with an 80 ms time frame. The ML models employed include: (a) SVM, (b) IBK, (c) S-SVM+, (d) S-IBK+. The plotted data represent 25% of the parent database that were not previously included in the training process of the ML models. The dashed line represents the line of ideality and the solid lines represent ±10% boundaries.

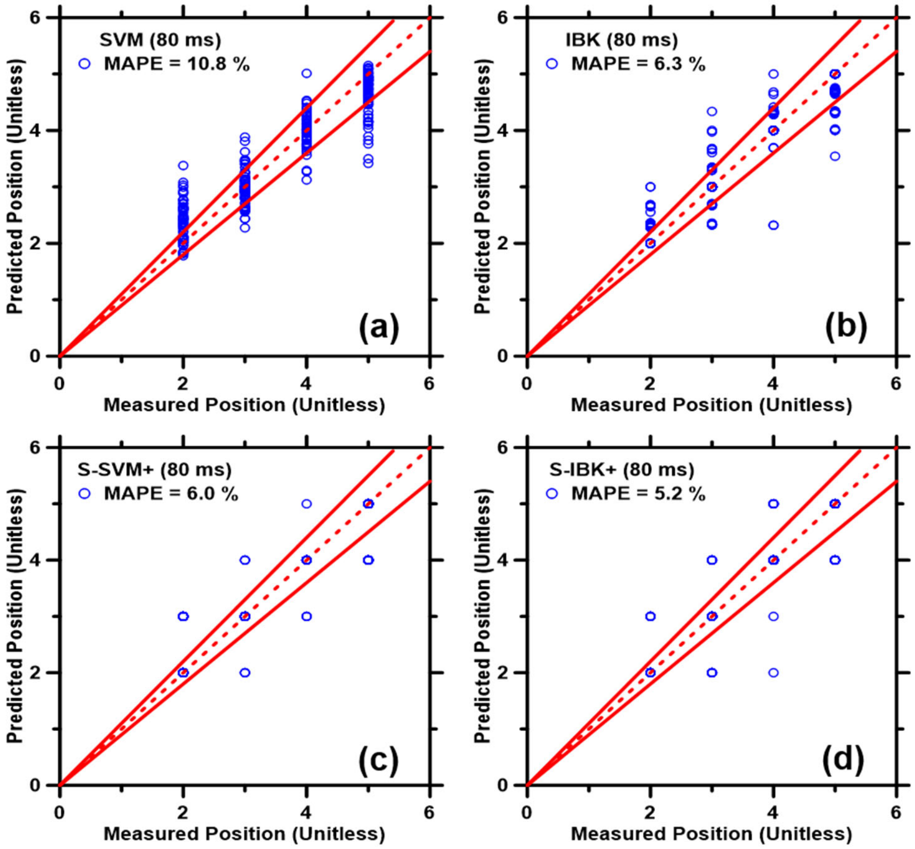

Figure S12 Graphs of predicted impact latitude versus measured latitude for the new training dataset with an 80 ms time frame. The ML models employed include: (a) SVM, (b) IBK, (c) S-SVM+, (d) S-IBK+. The plotted data represent 25% of the parent database that were not previously included in the training process of the ML models. The dashed line represents the line of ideality and the solid lines represent ±10% boundaries.

Table S7. Prediction performance on impact magnitude with the old (200 ms) and new (80 ms) training dataset, measured based the test set (i.e., 25% of the parent database). Five statistical parameters (i.e. R, R^2, MAE, MAPE, and RMSE) and the composite performance index (CPI) are shown.

ML Model	R<br>Unitless	R^2<br>Unitless	MAE<br>cm	MAPE<br>%	RMSE<br>cm	CPI<br>Unitless
SVM	0.891	0.793	3.492	8.991	4.730	0.120
**S-SVM+**	**0.889**	**0.790**	**2.980**	**7.635**	**4.680**	**0.000**
SVM (80 ms)	0.881	0.776	3.540	9.140	4.811	0.179
S-SVM+ (80 ms)	0.868	0.754	3.420	8.899	5.030	0.225
IBK	0.782	0.611	4.780	11.814	6.561	0.999
S-IBK+	0.782	0.611	4.540	11.570	6.332	0.937
IBK (80 ms)	0.845	0.714	4.283	10.740	5.535	0.551
S-IBK+ (80 ms)	0.809	0.655	4.360	10.917	6.033	0.753

Table S8. Prediction performance on impact direction with the old (200 ms) and new (80 ms) training dataset, measured based on the test set (i.e., 25% of the parent database). Five statistical parameters (i.e. R, R^2, MAE, MAPE, and RMSE) and the composite performance index (CPI) are shown.

ML Model	R<br>Unitless	R^2<br>Unitless	MAE<br>degree	MAPE<br>%	RMSE<br>degree	CPI<br>Unitless
SVM	0.960	0.921	21.415	24.554	30.781	0.845
S-SVM+	0.956	0.913	19.920	23.154	32.087	0.913
SVM (80 ms)	0.971	0.942	17.747	18.920	26.726	0.445
S-SVM+ (80 ms)	0.972	0.945	13.440	14.492	25.456	0.289
IBK	0.952	0.905	7.492	4.670	32.586	0.656
S-IBK+	0.971	0.942	3.960	3.536	25.385	0.082
IBK (80 ms)	0.960	0.921	7.930	5.870	29.483	0.448
**S-IBK+ (80 ms)**	**0.973**	**0.948**	**5.880**	**4.846**	**24.075**	**0.037**

Table S9. Prediction performance on impact latitude with the old (200 ms) and new (80 ms) training dataset, measured based on the test set (i.e., 25% of the parent database). Five statistical parameters (i.e. R, R^2, MAE, MAPE, and RMSE) and the composite performance index (CPI) are shown.

ML Model	R Unitless	R^2 Unitless	MAE Unitless	MAPE %	RMSE Unitless	CPI Unitless
SVM	0.924	0.855	0.323	10.853	0.440	0.839
S-SVM+	0.917	0.842	0.192	6.633	0.447	0.674
SVM (80 ms)	0.924	0.854	0.322	10.760	0.440	0.839
S-SVM+ (80 ms)	0.927	0.860	0.176	5.960	0.420	0.424
IBK	0.915	0.837	0.204	6.373	0.454	0.728
S-IBK+	0.927	0.859	0.140	4.600	0.424	0.361
**IBK (80 ms)**	**0.943**	**0.889**	**0.199**	**6.260**	**0.373**	**0.118**
S-IBK+ (80 ms)	0.928	0.862	0.168	5.193	0.420	0.375

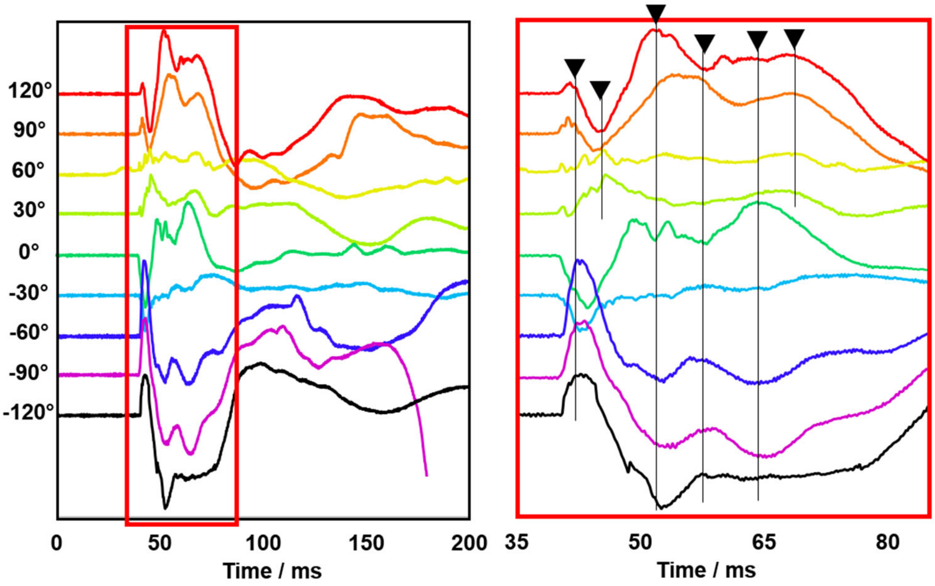

Figure S13 Transient oscillatory signals generated by nine different impact directions on the wireless smart helmet, conducted using the mid-level impact energy, 10.82 J. Full view of the first 200 ms transient signals is shown on the left. Expanded view of the red box is shown to the right illustrating the first 50 ms signals. Black arrows and lines indicate the signal patterns of peaks-and-valleys.

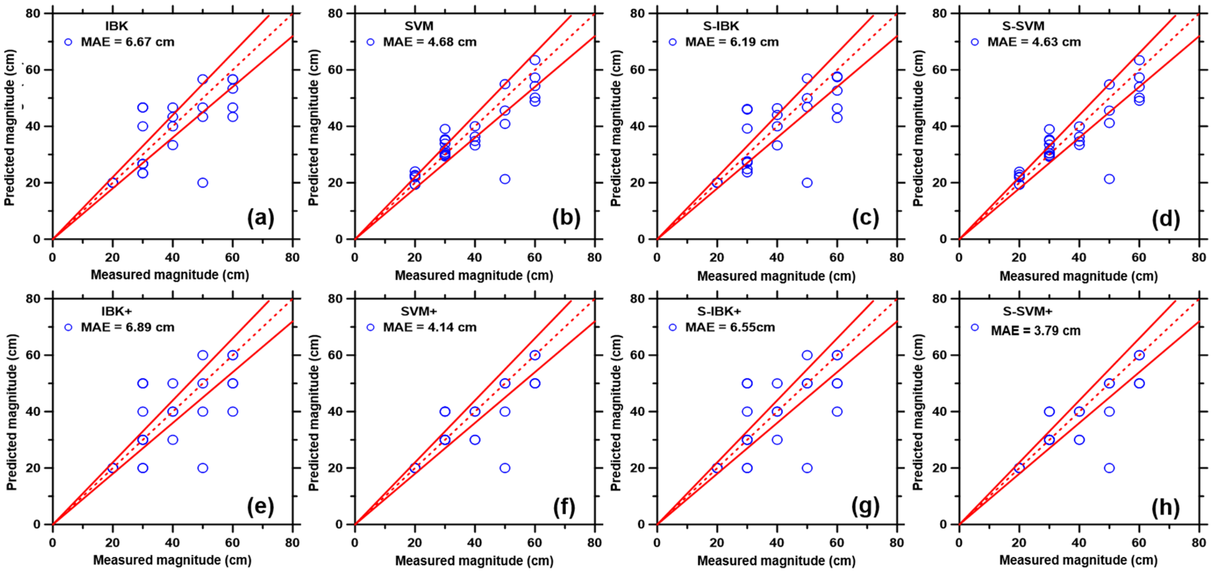

Figure S14 Graphs of predicted impact magnitudes versus measured magnitudes for the wireless sensing datasets derived from eight ML models. The ML models employed include: (a) IBK, (b) SVM, (c) S-IBK, (d) S-SVM, (e) IBK+, (f) SVM+, (g) S-IBK+, (h) S-SVM+. The plotted data represent 25% of the parent database that were not previously included in the training process of the ML models. The dashed line represents the line of ideality and the solid lines represent ±10% boundaries.

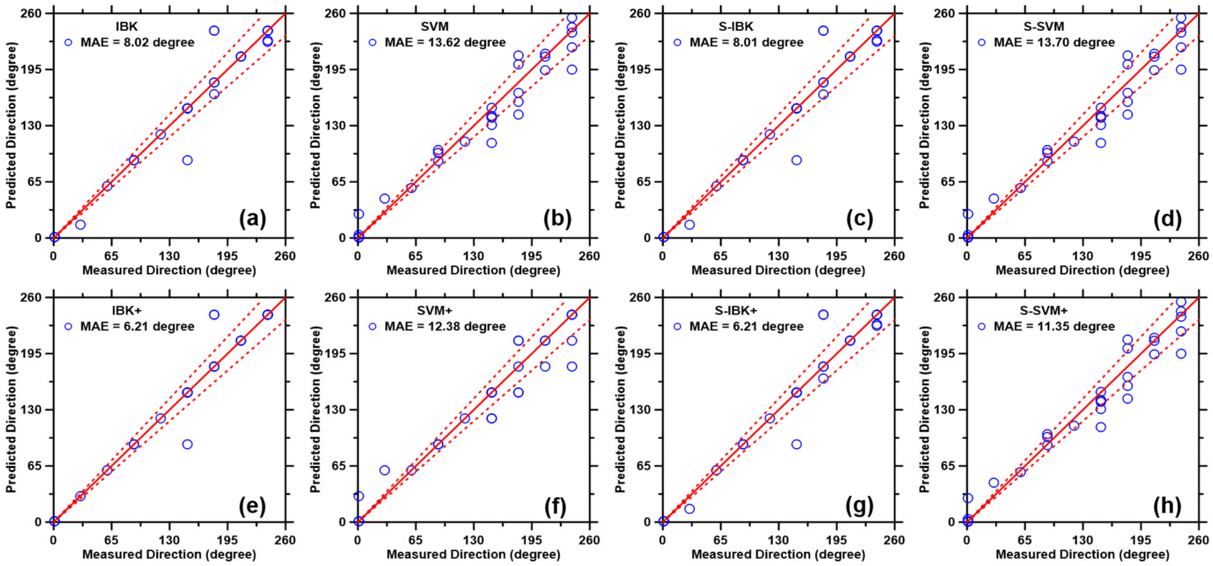

Figure S15 Graphs of predicted impact directions versus measured directions for the wireless sensing datasets derived from eight ML models. The ML models employed include: (a) IBK, (b) SVM, (c) S-IBK, (d) S-SVM, (e) IBK+, (f) SVM+, (g) S-IBK+, (h) S-SVM+. The plotted data represent 25% of the parent database that were not previously included in the training process of the ML models. The dashed line represents the line of ideality and the solid lines represent ±10% boundaries.

Table S10. Prediction performance on impact magnitude for the wireless sensing datasets, measured based the test set (i.e., 25% of the parent database). Five statistical parameters (i.e., R, R^2, MAE, MAPE, and RMSE) and the composite performance index (CPI) are shown. The results of the best performing ML models are highlighted in bold.

ML Model	R<br>Unitless	R^2<br>Unitless	MAE<br>cm	MAPE<br>%	RMSE<br>cm	CPI<br>Unitless
IBK	0.751	0.564	6.667	17.107	9.629	0.785
SVM	0.862	0.743	4.676	11.789	7.197	0.144
S-IBK	0.762	0.581	6.193	15.832	9.385	0.687
S-SVM	0.864	0.747	4.632	11.641	7.152	0.130
IBK+	0.699	0.488	6.897	17.816	10.828	1.000
SVM+	0.838	0.701	4.138	9.655	7.878	0.130
S-IBK+	0.711	0.506	6.552	17.126	10.667	0.927
**S-SVM+**	**0.852**	**0.726**	**3.793**	**8.506**	**7.656**	**0.071**

Table S11. Prediction performance on impact direction for the wireless sensing datasets, measured based on impact directions. Five statistical parameters (i.e., R, R^2, MAE, MAPE, and RMSE) and the composite performance index (CPI) are shown. The results of the best performing ML models are highlighted in bold.

ML Model	R<br>Unitless	R^2<br>Unitless	MAE<br>degree	MAPE<br>%	RMSE<br>degree	CPI<br>Unitless
IBK	0.970	0.941	8.016	5.971	19.908	0.579
SVM	0.973	0.947	13.622	113.205	18.439	0.626
S-IBK	0.970	0.941	8.013	5.972	19.907	0.578
S-SVM	0.974	0.948	13.702	113.624	18.376	0.609
**IBK+**	**0.973**	**0.946**	**6.207**	**3.678**	**19.298**	**0.340**
SVM+	0.977	0.955	12.379	108.912	20.795	0.556
**S-IBK+**	**0.973**	**0.946**	**6.207**	**3.678**	**19.298**	**0.340**
S-SVM+	0.973	0.947	11.345	108.481	18.421	0.554
